# Association between sow and piglet blood hemoglobin concentrations and stillbirth risk

**DOI:** 10.1186/s13028-019-0496-7

**Published:** 2019-12-10

**Authors:** Sheeva Bhattarai, Tore Framstad, Jens Peter Nielsen

**Affiliations:** 1 Department of Veterinary and Animal Sciences, Faculty of Health and Medical Sciences, University of Copenhagen, Grønnegårdsvej 2, 1870 Frederiksberg C, Denmark; 2 Department of Production Animal Clinical Sciences, Faculty of Veterinary Medicine, Norwegian University of Life Sciences, Campus Adamstuen, NO-0033 Oslo, Norway

**Keywords:** Fetus, Hemocue, Iron, Pig, Pregnancy, Stillborn

## Abstract

Previous studies have indicated that high piglet blood hemoglobin concentration (HbC) at birth lead to better performance later in life. The primary objective of this study was to investigate the association between sow and piglet blood HbC at farrowing. A secondary objective was to investigate the relationship between sow HbC and probability of stillbirths. Farrowings were observed in 22 sows in a Danish commercial herd. Maternal blood HbC was measured 1–3 days before farrowing using a HemoCue 201 + Hb device. Similarly, HbC was measured from 144 offspring piglets before colostrum intake. Total number of born piglets per litter and parity of the sow were recorded and stillborn piglets were identified using a lung flotation technique. The association between HbC in sows and piglets was determined using a linear mixed model. The relationship between sow HbC and probability of stillbirths was determined using a generalized linear model. The mean HbC of the sows and piglets were 106.9 ± 12.2 and 124.4 ± 19.9 g/L, respectively. A tendency towards a positive association between HbC of the piglets and HbC of the sow around farrowing was found (P = 0.058). Additionally, the probability of stillbirths was negatively associated with the sow HbC (P = 0.021). These results indicate that HbC in newborn piglets may be increased by increasing the sow HbC. Furthermore, stillbirth rates might be reduced by increasing the HbC of the sow.

## Findings

The iron supply of the pig fetus relies entirely on maternal supply [1]. Therefore, sufficient iron availability in sows is necessary to cope with the demands of the growing fetuses. Improved iron status and hemoglobin concentrations (HbC) at birth could have carry over effects on pre-weaning survival [2]. We have previously shown that piglets with higher HbC at weaning had higher average daily gain during the subsequent post-weaning period [3]. Furthermore, stillbirth in piglets is an economic, welfare and ethical problem related to high litter size. It has become a challenge to reduce stillbirth rates, as the underlying causes are not often obvious. Iron deficiency anemia as a cause of stillbirths in piglets has been reported previously [4, 5]. We have recently found that the probability of stillbirth in pigs decreases with increasing HbC in the sows [6]. These findings underline the importance of blood variables, which are related to iron availability of sows and their offspring. The primary objective of our study was therefore to investigate the association between sow and piglet HbC at farrowing. The secondary objective was to investigate the effect of sow HbC on the probability of stillbirths.

The study was a cohort carried out in a Danish herd with 1700 sows and 75 weekly farrowing. Farrowing was induced by intramuscular injection of prostaglandin by the herd veterinarian. A total of 22 sows and their 144 offspring piglets were included. Blood HbC was measured from the sows 1–3 days before farrowing and in piglets before colostrum intake. The HbC in the sows and piglets were analysed with a HemoCue 201 + Hb photometer (Hemocue) (AB Leo Diagnostics, Helsingborg, Sweden). The animal was fixed and the ear was cleaned. According to the manufacturer’s instructions, a microlancet was used to puncture one of the auricular veins of the sow or piglet. The first droplet of blood was removed and the second droplet was drawn into a microcuvette by capillary force. Every second piglet in the birth order (the 2nd, the 4th, etc.) was selected for blood sampling and this resulted in sampling of 2 to 9 piglets per sow. Farrowings were observed and those piglets were separated immediately from the sow until blood sampling. Stillborn or mummified fetuses were included in the birth order, but not sampled. Stillbirth, i.e. lack of air in the lung of an otherwise normal piglet, was verified using a lung flotation test [6] and was used to separate stillborn piglets from those which died just after farrowing. Number of total born piglets and parity of the sow were recorded after termination of farrowing. Mummified fetuses were not included in the number of born or stillborn piglets. Data analysis was performed using SAS 9.4 (SAS 9.4, SAS institute Inc, Cary, NC, USA).

Stillbirth rate was calculated as the number of stillborn piglets divided by the number of total born piglets per litter. Three parity ranks of sows were defined: rank 1 (parity 1), rank 2 (parities 2 and 3) and rank 3 (parity > 3). The birth order of the piglets was divided into four categories; Early (order 1–5), mid (order 6–10), late (order 11–15) and last (order > 15). The sows were divided into two categories based on their HbC, anemic (HbC < 103 g/L) and non-anemic (HbC ≥ 103 g/L). The cut off value for anemia was based on our recent study on reference intervals for sows [7].

The association between HbC of the sow and the piglets was determined using a linear mixed model with PROC MIXED procedure. HbC of the piglet was the response variable. Explanatory variables included the fixed effects of sow HbC, parity rank of the sow, birth order category of piglets and total number of piglets born per litter. Litter was included as a random effect.

The probability of stillbirth was analysed using a generalized linear model with PROC LOGISTIC procedure. HbC of sow, parity rank of the sow and total number of piglets born were the explanatory variables. Predicted probabilities of stillbirths were calculated for each level of sow HbC and plotted in a graph.

In both the models, an interaction between parity rank of the sow and total number of piglets born was also fitted. The variables were removed from the model using backward elimination. The level of statistical significance for all tests was set to P < 0.05 and P < 0.01 was regarded as a tendency.

The mean HbC of 22 sows and 144 piglets were 106.9 ± 12.2 and 124.4 ± 19.9 g/L, respectively. In total, 336 piglets were born with an average number of 16.1 ± 3. piglets per litter. The number of stillborn piglets was 22 resulting in a stillbirth rate of 6.5%. The average parity of sow was 3.1 ± 2.2. There were 4, 12 and 6 sows in parity rank 1, 2 and 3, respectively.

Nine sows were anemic. The percentage of stillborn piglets delivered by anemic sows was almost twice than in non-anemic sows (8.4 ± 10.1 vs 4.4 ± 4.4%).

**Fig. 1:**
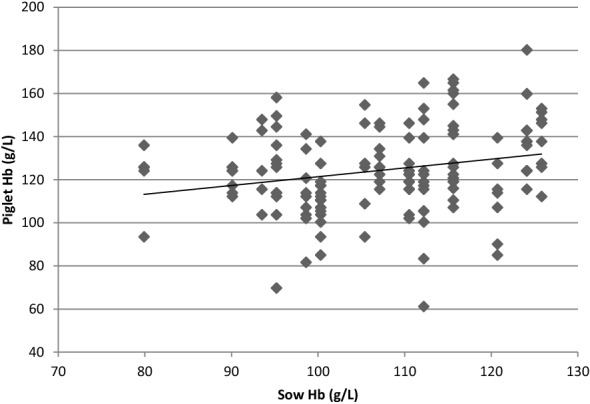
Correlation between blood hemoglobin concentration (HbC) in sows and piglets at farrowing (Note: each sow corresponded to 2 to 9 piglets) (correlation coefficient, r = 0.24). The association was tested on a linear mixed model [Intercept = 82.80, estimate for sow HbC (g/L) = 0.39 (P = 0.058)]

There was a positive correlation between sow HbC and piglet HbC (correlation coefficient, r = 0.24) (Fig. 1). The results of mixed model showed that there was a tendency of association between HbC of the piglet and the HbC of the sow (P = 0.058) with an estimate indication of 3.9 g/L increase in piglet HbC per 10 g/L increase in sow HbC.

**Fig. 2:**
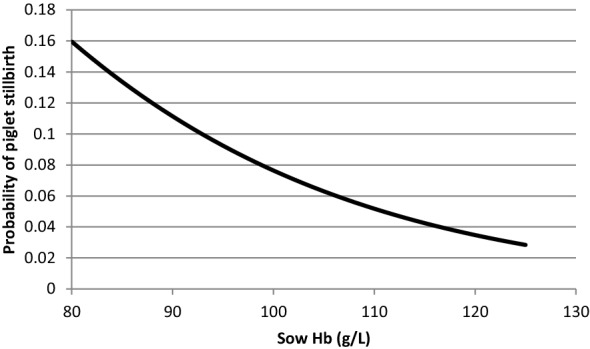
Probability of stillbirths in relation to sow hemoglobin concentration (HbC) at farrowing. Probability was estimated using estimate from the final model [Intercept = 1.65, probability estimate for sow HbC (g/L) = − 0.04 (P = 0.021)]

The risk of stillbirth was reduced with increased HbC of the sow (P = 0.021) (Fig. 2).

This study showed a tendency of positive association between HbC of the piglet at birth and HbC of the sow at farrowing. This indicates that increasing piglet HbC by increasing the HbC in the sow may be possible. It has been previously reported that piglets surviving until weaning had higher HbC at birth than the piglets that died before weaning [2]. This implies that high HbC at birth has a positive effect in increasing the post-natal survivability of piglets. The HbC at birth is particularly important in the period from birth until the piglet gets iron supplementation. A previous study [8] did not find any association between sow HbC and piglet HbC at birth measured neither three weeks before farrowing nor at the day of farrowing. Interestingly, another study had found a positive correlation between plasma ferritin concentrations in sows at late gestation and plasma ferritin in their new born offspring [9].

HbC in animals is closely related to iron availability. The transfer of iron through pig placenta is dependent on uteroferrin, an iron containing phosphatase [1]. It is produced by the endometrial cells in response to progesterone. Despite this mechanism, it has been suggested that the amount of placental iron transfer in pigs is low compared to other mammals [10]. Various animal and human studies have shown that the rate of iron transfer through the placenta depends on numerous factors including maternal iron status and species. It has also been shown that HbC is partly regulated by genetics in human and also in pigs [11, 12]. This may partly explain why maternal HbC and offspring HbC correlate.

This study used HemoCue for HbC measurement in sows and piglets. HemoCue is a convenient pen-side method of measuring HbC and several evaluations of its performance has been carried out in animal studies with varying results [13–18]. In sows and weaners, a very good correlation (correlation coefficient of 0.97 in each case) between HbC measurements from the standard hematology analyser and the HemoCue was found when jugular vein blood was used [16, 17].

Previously, we have established that the reference interval of HbC for mid-gestation Danish sows lies between 103 and 145 g/L [7]. We observed an average HbC of 106.9 g/L in the current study which is at the lower side of the established reference interval. However, the HbC of normal sows is generally decreasing from mid-gestation to farrowing because of increasing blood volumes. The average HbC of sows in this study was similar to that found in our previous published study (108.6 g/L) originating from the same herd but using a standard hematology analyser [6], although the group of sows differed. This indicates that HemoCue measurements are quite comparable to the measurements from analysers which are used as gold standards.

Interestingly, we also found a higher stillbirth rate in sows with low HbC at farrowing. This confirms the results of another study done in the same herd [6]. It is likely that a low oxygen capacity in the sow, due to a low HbC, could either increase the risk of piglets hypoxia during delivery [4] or decrease myometrial contractions [19], thereby causing stillbirths. Parity was not significant in this study which might be due to very few numbers of sows per parity ranks.

In conclusion, there was a tendency of positive association between HbC in pre-colostrum piglets at birth and HbC of the sow at farrowing. As the result was a tendency, it is difficult to draw firm conclusions based in this limited study. Also, a previously described association between sow HbC and stillbirth risk was confirmed. In the future, research focussing on improving the HbC of the sow would be worthwhile.

## Data Availability

The datasets used and/or analysed during the current study are available from the corresponding author on reasonable request.

## Prior publication

Data have not been published previously.
